# Association between dispensing of low-value oral albuterol and removal from Medicaid preferred drug lists

**DOI:** 10.1186/s12913-022-07955-x

**Published:** 2022-04-26

**Authors:** Anna Volerman, Alison Pelczar, Rena Conti, Christina Ciaccio, Kao-Ping Chua

**Affiliations:** 1 Department of Medicine, University of Chicago, 5841 S Maryland Avenue, MC 2007, Chicago, IL 60637 USA; 2 Department of Pediatrics, University of Chicago, Chicago, IL USA; 3 Harris School of Public Policy, University of Chicago (at time of project), Chicago, IL USA; 4 Department of Markets, Public Policy, and Law, Boston University Questrom School of Business, Boston, MA USA; 5 University of Michigan Medical School, Ann Arbor, MI USA

**Keywords:** Albuterol, Low-value care, Preferred drug list, Medicaid

## Abstract

**Background:**

Oral albuterol has worse efficacy and side effects compared with inhaled albuterol, and thus its use has been discouraged for decades. Drug inclusion or exclusion on formularies have been associated with reductions in low-value care. This study examines dispensing of oral albuterol and inclusion of oral albuterol on state Medicaid drug formularies--Preferred Drug Lists (PDLs). It also evaluates the association between removal of oral albuterol from the PDL and dispensing levels.

**Methods:**

This quasi-experimental study determined oral albuterol inclusion on PDLs and dispensing between 2011 and 2018, using Medicaid program websites and the State Drug Utilization Database. Using a difference-in-differences model, we examine the association between removal of oral albuterol from Arkansas’ Medicaid PDL in 2014 and dispensing of this drug through Medicaid, with Iowa as a control state. The outcome measure was the percent of all albuterol prescriptions that were for oral albuterol.

**Results:**

A total of 28 state Medicaid PDLs included at least one formulation of oral albuterol in 2018. In 2018, 179,446 oral albuterol prescriptions were dispensed to Medicaid beneficiaries nationally. Medicaid programs paid approximately $3.0 million for oral albuterol prescriptions in 2018. Removal of oral albuterol syrup from the Arkansas PDL in March 2014 was associated with a more rapid decline in dispensing compared with Iowa which maintained this medication on their PDL.

**Conclusions:**

Findings suggest that removal of low-value medications, such as oral albuterol, from PDLs may be one avenue by which state Medicaid programs can reduce wasteful spending while improving guideline-based care.

**Supplementary Information:**

The online version contains supplementary material available at 10.1186/s12913-022-07955-x.

## Background

Low-value services are those that fail to improve health or result in small health improvements relative to their cost [1, 2]. Use of low-value services is estimated to cost at least $75–100 billion annually [3] and is widespread among all populations, including children [4, 5], non-elderly adults [6], and Medicare beneficiaries [7]. Prior research suggests health insurer coverage and reimbursement policies, including drug inclusion or exclusion on formularies [8], can reduce low-value care and promote high-value care [9, 10]. These findings highlight the importance of carefully designing formularies so that they only include high-value drugs.

Most state Medicaid programs use preferred Drug Lists (PDLs), a type of formulary that lists of medications typically covered by Medicaid without prior authorization. Studies suggest that PDLs can impact prescribing of several types of medications, including cardiovascular drugs [11]. Other studies suggest that Medicaid managed care organizations can reduce drug spending by designing formularies that steer members toward drugs with a lower cost than those included in the state PDL, suggesting that effects of PDLs on spending and value may not always be positive [12, 13]. Despite the ability of PDLs to influence prescribing, few studies have examined the effect of removing drugs that are always low-value from PDLs. One study suggested that removal of methadone from PDLs can reduce prescribing of this drug for pain management, but methadone can be used both appropriately and inappropriately for analgesia [10].

One example of a drug that is always low-value is oral albuterol, an asthma quick-relief medication which is available in both tablet and syrup formulations. Compared with inhaled albuterol, oral albuterol has worse efficacy and side effects [14]. Clinical guidelines have explicitly dissuaded healthcare professionals from prescribing oral rescue medications for decades. The original asthma guidelines in the United States, published in 1991, recommend aerosol therapy over oral delivery due to the faster onset of relief, fewer adverse effects, and ability to achieve the same relief with a lower dose [15]. The two guideline updates (1997 and 2007) make no mention of oral beta_2_-agonist medications as treatment options [16, 17]. Similarly, international guidelines either explicitly do not recommend oral albuterol or make no mention of it [18, 19].

Despite the absence of oral albuterol in guidelines and calls to cease prescribing of this medication [20], a 2006 study found that this drug was frequently used [21]. In this study, we report more recent data on dispensing of oral albuterol to Medicaid patients, assess the inclusion of oral albuterol in Medicaid PDLs, and evaluate the association between the removal of oral albuterol from Arkansas’ PDL and dispensing of this drug.

## Methods

This quasi-experimental study examines oral albuterol dispensing and Medicaid PDLs between 2011 and 2018.

### Data source for albuterol dispensing

We obtained data on albuterol prescription dispensing to Medicaid enrollees in all states between 2011 and 2018 from the State Drug Utilization Database, which is maintained by the Centers for Medicare & Medicaid Services [22]. This database reports the quarterly number of prescriptions dispensed to Medicaid enrollees by national drug code (NDC). We identified products containing albuterol using a list of NDCs obtained from IBM Micromedex RED BOOK (see Table E1 in Online Supplement for full list) [23]. Our list included all albuterol NDC codes that were active during the study period.

In the State Drug Utilization Database, data are suppressed for any quarterly count less than 11. We imputed suppressed counts in the same manner as prior studies using the same dataset [24]. This method leveraged the discrepancy between the sum of state-level totals and the national total. Imputed counts were weighted inverse to the number of quarters during the year that the count was suppressed, so that counts less frequently suppressed received greater weight (see Methods E1 in Online Supplement for description of the full methodology). When suppressed cells were dropped instead of imputed, results from difference-in-differences analyses were unchanged (see Table E2 in Online Supplement, which contains the results without imputed counts)**.**

### Data source for PDLs

For the analysis of oral albuterol inclusion on 2018 PDLs, data on PDLs were obtained from Medicaid program websites. If PDLs were unavailable on websites, published minutes and notes for the state’s Pharmacy and Therapeutics Committee were reviewed for indications of changes. If neither was available, the state’s Medicaid office was contacted to clarify coverage or obtain prior PDL versions. When more than one list was published during 2018, all versions were reviewed to evaluate whether changes in coverage occurred. An albuterol formulation was considered preferred if it was included on the PDL; otherwise, it was considered non-preferred.

The analysis was limited to PDLs for FFS plans. Four states (Hawaii, New Jersey, New Mexico, and South Dakota) did not use PDLs for their FFS plans in 2018 [25] and were excluded, leaving 46 states and the District of Columbia (henceforth referred to as “states”). Of the 47 states, 11 had FFS only (no managed care organization (MCO) plans) in 2018, 13 had both FFS and MCO plans and used the same PDL for both (uniform PDL), and 23 had both FFS and MCO plans but used different PDLs [25]. For these 23 states, the PDLs for MCO plans were not examined.

For each of the 47 states using PDLs in 2018, we determined whether at least one formulation of oral albuterol was considered preferred on the state’s 2018 FFS PDL. Additionally, we calculated total reimbursement for oral albuterol prescriptions across all state Medicaid programs in 2018. We conducted these analyses to determine the degree to which Medicaid programs continue to include low-value oral albuterol on PDLs and to estimate the direct amount of wasteful spending on this drug by Medicaid programs.

### Selection of states for difference-in-differences analysis

Nineteen states did not consider any form of oral albuterol as preferred on their FFS PDL in 2018. For these states, PDLs from 2011 to 2018 were reviewed to determine whether any oral albuterol formulations had changed from preferred to non-preferred status. Changes were identified in six states (2012: Idaho; 2016: Connecticut, Delaware; 2017: Michigan; 2018: Kentucky, New Jersey, North Carolina). Three states were not considered, because PDL removal occurred too early or late during the study period to have sufficient pre-intervention or post-intervention data. Two states (Connecticut and Delaware) were not considered because dispensing totals of oral albuterol in the quarter before PDL removal were 0.1–0.3%, levels that were too low to measure impact. In contrast, dispensing totals before PDL removal were high enough to measure impact in Arkansas, which changed oral albuterol syrup from preferred to non-preferred in March 2014, midway through the study period.

To identify potential control states for Arkansas, the percentage of dispensed albuterol prescriptions that were for oral albuterol syrup in 2011 was calculated for each state. This percentage was used because it is unaffected by the number of Medicaid enrollees, in contrast to raw prescription counts. States with a similar percentage of dispensed albuterol prescriptions that were for oral albuterol syrup to Arkansas in 2011 were considered. Iowa was chosen as the control state for several reasons. First, the trends for this percentage during the pre-intervention period between 2011 and 2013 were parallel to Arkansas. Second, Iowa did not experience any changes in albuterol coverage on its PDL between 2011 and 2018. Third, while Iowa transitioned from being fee-for-service only to a combination of fee-for-service and managed care organization plans in April 2016, the state utilized a uniform PDL that applied to both types of plans throughout the study period. Finally, Iowa expanded Medicaid under the Affordable Care Act in January 2014, as did Arkansas.

### Statistical analysis

To examine the association between removal of oral albuterol syrup from Arkansas’ PDL in 2014 and dispensing of this drug, a difference-in-differences analysis was performed using data between 2011 and 2018. The pre-intervention period consisted of 12 quarters between January 2011 and December 2013, and the post-intervention period included 19 quarters between April 2014 and December 2018. The quarter containing March 2014 was excluded. A second difference-in-differences model including only data through 2015 was also estimated, as Iowa started to use MCOs in 2016, while Arkansas had only FFS plans from 2011 to 2018.

The dependent variable was the percentage of albuterol prescriptions dispensed that were for oral albuterol syrup. The model included indicators for quarter to adjust for seasonal patterns in dispensing. Robust standard errors were used.

To test the parallel trends assumption, a linear regression model was fit using only data from 2011 to 2013. Terms included time as a continuous variable, an indicator for Arkansas, and their interaction.

## Results

### Oral albuterol dispensing

In 2018, the database indicated that 174,505 oral albuterol prescriptions were dispensed to Medicaid beneficiaries nationally. After imputation, we estimate that the true total was 179,466 (see Table 1 for prescriptions by state). This total included 10,736 prescriptions for immediate-release tablets, 3182 for extended-release tablets, and 165,528 for syrup. Based on reimbursement data for the 174,505 non-imputed prescriptions in the database, Medicaid programs paid $3.0 million for oral albuterol in 2018. In 2018, the percentage of albuterol prescriptions across all state Medicaid programs that were for oral albuterol was 0.7%, down from 3.2% in 2011.

**Table 1:** Oral albuterol inclusion status on Medicaid fee-for-service preferred drug lists (PDLs) and oral albuterol prescriptions by state in 2018^1^

State	Status of albuterol on PDL		Oral albuterol prescriptions	
	Tablet preferred^**1**^	Syrup preferred	Number	Percent of all prescriptions
Alabama	x	x	3266	1.17
Alaska	x	x	31	0.07
Arizona		x	1381	0.29
Arkansas			0	0.00
California	x	x	43,831	1.54
Colorado			425	0.16
Connecticut		x	428	0.13
Delaware		x	56	0.07
District of Columbia		x	32	0.04
Florida		x	5596	0.47
Georgia			7141	1.03
Hawaii	*	*	2654	2.53
Idaho			20	0.03
Illinois		x	7000	0.77
Indiana		x	2770	0.56
Iowa	x	x	269	0.20
Kansas			250	0.21
Kentucky			2504	0.41
Louisiana		x	5264	1.13
Maine			106	0.13
Maryland		x	2097	0.45
Massachusetts	x	x	673	0.13
Michigan		x	9179	1.01
Minnesota		x	384	0.12
Mississippi	x		3377	1.99
Missouri			940	0.26
Montana			47	0.06
Nebraska		x	122	0.16
Nevada			1025	0.43
New Hampshire			108	0.22
New Jersey	*	*	7251	1.01
New Mexico	*	*	1947	1.06
New York			31,776	1.42
North Carolina^2^	x	x	1810	0.34
North Dakota	x	x	56	0.26
Ohio			3235	0.25
Oklahoma			1140	0.48
Oregon			269	0.10
Pennsylvania			2327	0.21
Rhode Island			64	0.06
South Carolina	x	x	1205	0.49
South Dakota	*	*	35	0.17
Tennessee	x	x	2019	0.47
Texas		x	19,356	1.34
Utah	x	x	308	0.51
Vermont	x	x	27	0.06
Virginia			2700	0.69
Washington	x	x	660	0.15
West Virginia	x		1762	0.68
Wisconsin	x	x	416	0.11
Wyoming			97	0.62
National	15	26	179,446	0.78

^1^For albuterol tablets, states may have considered immediate-release and/or extended-release tablets to be preferred. These formulations were categorized separately, as some states considered one to be preferred and the other to be non-preferred. Table shows states that had at least one formulation of tablets as preferred

^2^Oral albuterol tablets were removed from the PDL in North Carolina in December 2018

*States with no preferred drug lists

### Inclusion of albuterol on PDLs

In 2018, 28 of the 47 states that used PDLs included at least one oral albuterol formulation on their PDL. Of these 28 states, 26 had preferred status for oral albuterol syrup and 15 had preferred status for at least one formulation of oral albuterol tablet (immediate-release or extended-release) (Table 1).

### Removal of albuterol syrup from Arkansas PDL

In Arkansas, oral albuterol syrup comprised an average 0.93% of all albuterol prescriptions dispensed per quarter under Medicaid during 2011–2013, compared to 0.83% in Iowa. This percentage declined over time in both states. Rates of decline were similar (*p* = 0.50), suggesting the parallel trends assumption was met.

**Fig. 1:**
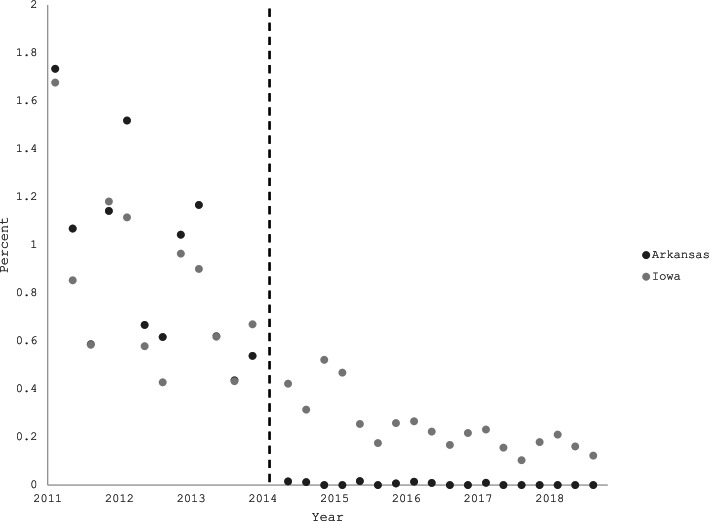
Percent of dispensed albuterol prescriptions that were for oral albuterol syrup prescriptions in Iowa and Arkansas, by quarter. This percentage dropped sharply in Arkansas in quarter 2 of 2014, following the removal of oral albuterol from the Arkansas Medicaid preferred drug list in March 2014. A sharp drop is not observed in Iowa

After Arkansas removed albuterol syrup from the PDL in March 2014, the percent of dispensed albuterol prescriptions that were for albuterol syrup almost immediately declined to levels near zero, while this percentage declined more slowly in Iowa (Fig. 1). PDL removal was associated with a 0.33 percentage-point greater decline in this percentage in Arkansas compared with Iowa (*p* = 0.01) (Table 2). Restricting the time period to 2011–2015 produced similar results (differential decrease of 0.43 percentage points, *p* = 0.02).

**Table 2:** Results from difference-in-differences analysis examining association between dispensing and removal of oral albuterol syrup from Arkansas’ PDL in March 2014

	2011–2018		2011–2015 (Sensitivity Analysis)	
	Estimate (SE)	*p*	Estimate (SE)	*p*
Intercept	1.04 (0.12)	< 0.001	1.19 (0.14)	< 0.001
Difference in the pre-intervention period	0.09 (0.12)	0.43	0.09 (0.10)	0.37
Change during post-intervention period	−0.58 (0.08)	< 0.001	−0.44 (0.09)	< 0.001
Additional change during post-intervention period in Arkansas	−0.33 (0.13)	0.01	−0.43 (0.15)	0.01

*SE* standard error

Dependent variable is percentage of albuterol prescriptions that were for oral albuterol syrup

## Discussion

We provide an updated snapshot of the prescribing of oral albuterol and report on its inclusion on the majority of state PDLs in 2018 despite its worse efficacy and side effect profile compared with inhaled albuterol. Moreover, we provide quasi-experimental evidence that removal of oral albuterol from the PDL in Arkansas was associated with an immediate near-elimination of dispensing of this drug. In contrast, dispensing of oral albuterol still occurred at the end of the study period in Iowa, which did not remove oral albuterol from its PDL. Findings suggest state Medicaid programs can reduce wasteful spending and potentially improve patient health by excluding low-value drugs from PDLs.

Oral albuterol has not been recommended as part of clinical guidelines for asthma for over three decades. Despite evidence-based recommendations in place, this study demonstrates that it continues to be utilized with over 175,000 prescriptions filled in 2018. Total spending on these prescriptions in 2018 was a modest $3.0 million, representing a fraction of total Medicaid spending for inhaled albuterol in 2018 ($1.1 billion for 22,882,125 prescriptions) [26]. However, this amount only represents the direct costs of oral albuterol dispensing. The true cost is likely higher, as the use of oral albuterol over more effective inhaled formulations may lead to avoidable and costly emergency department visits for asthma exacerbations or potentially symptoms related to the side effects of oral albuterol [27]. Consequently, potential savings if Medicaid programs removed oral albuterol from PDLs may be greater than just the direct costs of dispensing.

Formulary placement, including PDL inclusion, can be a tool to promote use of high-value care, as seen with curative hepatitis C treatment [28]. Conversely, PDL removal can be used to contain costs. Although studies have documented decreases in dispensing following removal of several expensive drugs from PDLs [9, 10], our study provides some of the first evidence that PDL removal can reduce dispensing of a drug that is universally low-value.

While this study focused on oral albuterol, findings may be applicable to other low-value drugs. An analysis of employer pharmacy benefits demonstrated that reducing the use of high-cost, low-value drugs could lead to an annual savings of 3–24% of the overall pharmacy spending [29]. For example, some formularies include branded combination products that do not necessarily provide sufficient clinical value to justify their higher cost compared with their individual generic components, such as ibuprofen/famotidine (Duexis) [30].

This study has several limitations. First, in the Medicaid dispensing database, there may be some lag between when a prescription is dispensed and when it was reported. In addition, data sources did not report why Arkansas removed oral albuterol from its PDL. Consequently, it is unclear whether the resulting reductions in dispensing were intended or not. Second, the aggregated nature of the data precluded us from determining whether oral albuterol prescriptions were replaced with inhaled albuterol prescriptions among Medicaid enrollees in Arkansas. Third, both Arkansas and Iowa adopted Medicaid expansion in January 2014, two months before the PDL change in Arkansas. However, we have no a priori reason to suspect that Medicaid expansion would differentially cause shifts in the percentage of albuterol prescriptions that were for oral albuterol between the two states. Finally, the suppression of small cells in this database meant that imputation was necessary; we show that this did not significantly impact results.

## Conclusion

Medicaid PDL removal may be a powerful tool for reducing dispensing of low-value drugs to Medicaid recipients. Moreover, the importance of this tool may only be increasing as a greater number of states carve out prescription drug benefits from managed care organization capitation payments and adopt uniform PDLs that apply both to fee-for-service and managed care organization enrollees [31]. State Medicaid programs and insurers more broadly should consider the removal low-value drugs from formularies and PDLs in order to reduce wasteful spending and potentially improve patient health by decreasing use of medications that have more effective alternatives.

## Supplementary Information

**Additional file 1.**

**Additional file 2.**

## Data Availability

All data generated or analysed during this study are included in this published article and its supplementary information files.

## Abbreviations

FFS: Fee for service
MCO: Managed Care Organization
NDC: National Drug Code
PDL: Preferred drug list
