# Supplementary material for: Association between dispensing of low-value oral albuterol and removal from Medicaid preferred drug lists
Source: BMC Health Serv Res. 2022 Apr 26;22:562. doi: 10.1186/s12913-022-07955-x (PMC9044602; doi:10.1186/s12913-022-07955-x)
Supplement: Supplementary file 1 — Additional file 1. [file 12913_2022_7955_MOESM1_ESM.docx]

**Online Supplement**

**Title:** Association between Dispensing of Low-Value Oral Albuterol and Removal from Medicaid Preferred Drug Lists

**Authors:** Anna Volerman, MD, Alison Pelczar, MPP, Rena Conti, PhD, Christina Ciaccio, MD, MSc, Kao-Ping Chua, MD, PhD

**Table E1:** National Drug Codes (NDCs) for albuterol dispensed in 2018

| **NDC** | **Form** | **Route** |
| --- | --- | --- |
| 00378-4122-01 | Extended Release Tablet | Oral |
| 00378-4124-01 | Extended Release Tablet | Oral |
| 68774-0400-01 | Extended Release Tablet | Oral |
| 68774-0600-01 | Extended Release Tablet | Oral |
| 50383-0740-16 | Syrup | Oral |
| 00603-1008-58 | Syrup | Oral |
| 00472-0825-16 | Syrup | Oral |
| 00093-0661-16 | Syrup | Oral |
| 66689-0100-16 | Syrup | Oral |
| 64980-0442-01 | Tablet | Oral |
| 64980-0443-01 | Tablet | Oral |
| 69543-0291-10 | Tablet | Oral |
| 69543-0290-18 | Tablet | Oral |
| 69543-0290-10 | Tablet | Oral |
| 68084-0949-25 | Tablet | Oral |
| 68084-0949-95 | Tablet | Oral |
| 68084-0952-25 | Tablet | Oral |
| 69238-1344-01 | Tablet | Oral |
| 69238-1345-01 | Tablet | Oral |
| 51079-0657-20 | Tablet | Oral |
| 51079-0658-20 | Tablet | Oral |
| 00378-0572-05 | Tablet | Oral |
| 00378-0572-01 | Tablet | Oral |
| 00378-0255-05 | Tablet | Oral |
| 00378-0255-01 | Tablet | Oral |
| 53489-0176-01 | Tablet | Oral |
| 53489-0176-05 | Tablet | Oral |
| 53489-0177-01 | Tablet | Oral |
| 53489-0177-05 | Tablet | Oral |
| 59310-0580-20 | Powder | Inhalation |
| 49502-0692-03 | Solution | Inhalation |
| 49502-0693-03 | Solution | Inhalation |
| 00472-0831-23 | Solution | Inhalation |
| 50383-0741-20 | Solution | Inhalation |
| 49502-0697-61 | Solution | Inhalation |
| 49502-0697-29 | Solution | Inhalation |
| 49502-0697-24 | Solution | Inhalation |
| 00378-8270-91 | Solution | Inhalation |
| 00378-8270-93 | Solution | Inhalation |
| 00378-8270-55 | Solution | Inhalation |
| 00378-8270-52 | Solution | Inhalation |
| 00378-6992-52 | Solution | Inhalation |
| 00378-6991-52 | Solution | Inhalation |
| 00378-6990-93 | Solution | Inhalation |
| 00378-6990-91 | Solution | Inhalation |
| 00378-6990-58 | Solution | Inhalation |
| 00378-6990-52 | Solution | Inhalation |
| 00487-9501-25 | Solution | Inhalation |
| 00487-9501-01 | Solution | Inhalation |
| 00487-9501-02 | Solution | Inhalation |
| 00487-9501-03 | Solution | Inhalation |
| 00487-9501-60 | Solution | Inhalation |
| 00487-9901-02 | Solution | Inhalation |
| 00487-9901-30 | Solution | Inhalation |
| 00487-9904-01 | Solution | Inhalation |
| 00487-9904-02 | Solution | Inhalation |
| 00487-9904-25 | Solution | Inhalation |
| 00487-0301-01 | Solution | Inhalation |
| 00487-0301-02 | Solution | Inhalation |
| 76204-0200-01 | Solution | Inhalation |
| 76204-0200-25 | Solution | Inhalation |
| 76204-0200-30 | Solution | Inhalation |
| 76204-0200-60 | Solution | Inhalation |
| 00054-8063-11 | Solution | Inhalation |
| 66794-0001-30 | Solution | Inhalation |
| 00781-7155-64 | Solution | Inhalation |
| 00781-7155-29 | Solution | Inhalation |
| 00781-7155-86 | Solution | Inhalation |
| 00591-3797-30 | Solution | Inhalation |
| 00591-3797-60 | Solution | Inhalation |
| 00591-3797-83 | Solution | Inhalation |
| 00591-3467-53 | Solution | Inhalation |
| 00591-3468-53 | Solution | Inhalation |
| 00172-6405-49 | Solution | Inhalation |
| 24208-0347-20 | Solution | Inhalation |
| 16252-0097-33 | Solution | Inhalation |
| 16252-0097-22 | Solution | Inhalation |
| 16252-0097-66 | Solution | Inhalation |
| 00085-1336-01 | Solution | Inhalation |
| 00085-1806-01 | Solution | Inhalation |
| 59310-0579-22 | Suspension | Inhalation |
| 00085-1132-01 | Suspension | Inhalation |
| 00085-1132-04 | Suspension | Inhalation |
| 00173-0682-21 | Suspension | Inhalation |
| 00173-0682-20 | Suspension | Inhalation |
| 00173-0682-24 | Suspension | Inhalation |

**Table E2.** Results from difference-in-differences analysis examining association between dispensing and removal of oral albuterol syrup from Arkansas’ preferred drug list in March 2014, excluding imputed counts

|  | 2011-2018 | | 2011-2015 | |
| --- | --- | --- | --- | --- |
|  | Estimate (SE) | *p* | Estimate (SE) | *p* |
| Intercept | 1.04 (0.12) | <0.001 | 1.19 (0.14) | <0.001 |
| Difference in the pre-intervention period | 0.10 (0.12) | 0.43 | 0.10 (0.10) | 0.37 |
| Change during post-intervention period | -0.60 (0.09) | <0.001 | -0.44 (0.10) | <0.001 |
| Additional change during post-intervention period in Arkansas | -0.32 (0.13) | 0.02 | -0.43 (0.15) | 0.01 |

SE: standard error

**Methods E1:** Methodology for imputing suppressed prescription counts

In the State Drug Utilization Database maintained by the Centers for Medicare & Medicaid Services (CMS), all cell counts less than 11 are suppressed. Any necessary secondary suppression is also applied. Suppressed counts were imputed in a manner similar to other studies using the same dataset.^43^ The imputation process leveraged the discrepancies between the reported state totals and national totals. Imputation eliminated misleading zeroes without significantly altering nonzero percentages. The imputation process consisted of the following steps:

1. The frequency of suppression or a total of zero was determined at the most granular level possible (by NDC, state, quarter, and fee-for-service or managed care).
2. Imputation weights were assigned according to the suppression frequency: four for one quarter suppressed, three for two quarters, two for three quarters, and one for four quarters suppressed.
3. The discrepancy in albuterol prescriptions between the reported state totals and the reported national total was calculated, again at the most granular level possible.
4. This difference was divided proportionally according to imputation weight, and the imputed counts were rounded to the nearest integer.
5. For totals suppressed at the national level, the state-level imputation weight was imputed as the state-level total, and the sum of these weights was imputed as the national total.
